# Supplementary material for: WordCommentsAnalyzer: A windows software tool for qualitative research
Source: F1000Res. 2018 Sep 4;7:536. Originally published 2018 May 3. [Version 2] doi: 10.12688/f1000research.14819.2 (PMC6137411; doi:10.12688/f1000research.14819.2)
Supplement: Supplementary file 3 [file f1000research-7-17614-s0002.tgz › b2a79496-4ada-47c4-9682-a99ce867836d.zip!/Eight Iranian Health Journals/Yafteh _6.docx]

© Yafteh  . This work is licensed under CC BY 4.0 (https://creativecommons.org/licenses/by/4.0/).

Volume & Issue: دوره 18 - شماره 4

Title: بررسی و شناسایی مهمترین عوامل مؤثر بر خطاهای گفتاری در جامعه پرستاران

http://yafte.lums.ac.ir/article-1-2404-fa.html

مقدمه: یکی از موضوعات علم روانشناسی زبان، بررسی خطاهای گفتار است. خطاهای گفتار یا لغزش زبان امری طبیعی است که برای هر فردی اتفاق می­ افتد. پژوهش حاضر از آن جهت اهمیت دارد که پرستاری شغل حساس و مهمی است و خطاهای گفتاری ممکن است باعث اشتباه در درمان بیماران شود، ولی متأسفانه تاکنون تحقیقی در این زمینه انجام نشده است. این پژوهش به منظور بررسی تأثیر سه عامل شخصیت، استرس، خستگی و کم‌خوابی در ایجاد خطاهای گفتاری در پرستاران انجام شده است. مواد و روش ­ها: پژوهش حاضر از نوع توصیفی- تحلیلی می­ باشد. روش گردآوری داده ­ها به‌صورت میدانی بوده، نمونه­ های تحقیق را پرستاران شاغل در بیمارستان‌های شهر ایلام تشکیل داده‌اند. ابزار گردآوری داده­ ها پرسشنامه بوده و داده ­ها با استفاده از پرسشنامه ­های اطلاعات جعمعیت شناختی، شخصیت چند جنبه‌ای مینه­سوتا، پرسشنامه ویژگی‌های شخصیتی نئو و پرسشنامه­ های استرس شغلی جمع‌آوری شدند و برای تحلیل شاخص­ های آمار توصیفی و استنباطی داده­ها از نرم­افزار 20SPSS  استفاده شد. یافته­ ها: 30 نفر (60%) از پرستاران شرکت‌کننده در پژوهش زن و 19 نفر (38%) مرد بودند. یافته ­ها نشان داد که هر سه عامل نوع شخصیت، استرس و خستگی تأثیر مثبت و معناداری بر روی خطاهای گفتاری پرستاران دارند. از میان سه عامل مورد بررسی، عامل خستگی و کم‌خوابی بیشترین تأثیر را داشته است. بحث و نتیجه ­گیری: نتایج نشان داد که بین نوع شخصیت، استرس، خستگی و خطاهای گفتار ارتباط معناداری وجود دارد و توجه به عوامل ایجاد خطاهای گفتاری در پرستاران و تلاش برای کاهش و بهبود این عوامل ضروری است. لذا بایستی تلاش شود که با کاهش میزان خستگی و استرس پرستاران از طریق کاهش ساعات کاری و کنترل عوامل استرس­زا از شیوع این اختلالات و تأثیر منفی آن بر روند درمان جلوگیری به عمل آید.

Title: کفایت همودیالیز در بیماران همودیالیزی مزمن بیمارستان شهدای عشایر

http://yafte.lums.ac.ir/article-1-2405-fa.html

مقدمه: بیماری مزمن کلیه یک مشکل مهم سلامت است که شیوع آن در دنیا و در کشور ما در حال افزایش است. در مرحله نهایی بیماری کلیه بیمار مادام‌العمر نیازمند درمان جایگزین عملکرد کلیه خود با همودیالیز (شایع‌ترین)، دیالیز صفاقی و یا پیوند کلیه است. کفایت همودیالیز و وضعیت تغذیه‌ای این بیماران با میزان مرگ و میر آنها مرتبط است. مواد و روش ها: مطالعه‌ای توصیفی که در آن بیماران دچار نارسایی مزمن کلیه مراجعه کننده به بخش دیالیز بیمارستان شهدای عشایر با حداقل سه ماه سابقه دیالیز بررسی شدند. نمونه‌های خون و ادرار تهیه گردید و تمامی نمونه‌ها در یک آزمایشگاه و توسط یک نفر بررسی شدند. کفایت همودیالیز Kt/V و میزان کاتابولیسم پروتئین nPCR برای بیماران محاسبه گردید. داده‌ها توسط نرم افزار 16 SPSS  در سطح معنی داری 05/0 تحلیل شدند. یافته­ ها: از 79 بیمار بررسی شده 52 نفر (8/65 درصد) مذکر و 27 نفر (2/34 درصد) مؤنث بودند. میانگین سن بیماران حدود 62 سال بود. میانگین کفایت همودیالیز و میزان کاتابولیسم پروتئین بیماران به ترتیب 2/1 و 83/1 بود. کفایت همودیالیز با جنس فرد، سن، BMI و دیابتی بودن ارتباط داشت. میزان کاتابولیسم پروتئین در هفتاد بیمار (6/88 درصد) برابر یا بیش از 2/1 مشاهده شد. بحث و نتیجه ­گیری: در این مطالعه کفایت همودیالیز در زنان بهتر از مردان مشاهده شد که احتمالاً با اندازه جثه آنان ارتباط دارد. کفایت همودیالیز افراد مسن و یا چاق کمتر از افراد جوان‌تر یا کم وزن‌تر بود که احتمالاً ناشی از کاهش توده عضلانی و کاهش تولید کراتینین در آن‌هاست. میزان کاتابولیسم پروتئین در اکثریت بیماران در حد قابل قبول بود. برای افزایش کفایت همودیالیز، افزایش مدت زمان هر جلسه دیالیز، افزایش روزهای دیالیز در هفته و پیشگیری و رفع گردش خون مجدد توصیه می‌شود.

Title: تأثیر عصاره هیدرو الکلی پوست انار و ویتامین E بر میزان نیتریک اکسید و فعالیت آنزیمهای پاراکسوناز و میلوپراکسیداز در رتهای تحت تنش اکسیداتیو ورزش درمانده ساز

http://yafte.lums.ac.ir/article-1-2407-fa.html

مقدمه: مطالعات نشان داده‌اند که تولید رادیکال‌های آزاد در اثر تمرینات سنگین ورزشی بخصوص گونه اکسیژن (ROS) باعث آسیب به بافت‌ها شده که می‌توان با مصرف به‌موقع مواد آنتی اکسیدانی از این امر جلوگیری کرد. هدف مطالعه حاضر بررسی اثرات آنتی اکسیدانی عصاره هیدروالکلی پوست انار بر کاهش اثرات تنش اکسیداتیو ناشی از یک جلسه ورزش درمانده ساز بود. مواد و روش ­ها: در این مطالعه تعداد 32 سر موش صحرایی نر نژاد ویستار به‌طور تصادفی به چهار گروه هشت‌تایی تقسیم شدند: 1)con (گروه کنترل). 2) PPHE200 (mg/kg 200 عصاره هیدروالکلی پوست انار)، 3)PPHE250 (mg/kg 250 عصاره هیدروالکلی پوست انار) و 4) vit.E (mg/kg 5 ویتامین E). حیوانات به مدت 8 هفته به‌طور منظم روزانه یک ساعت ورزش داده شدند و در آخرین جلسه تمرین تحت استرس ورزش درمانده ساز قرار گرفتند. بلافاصله پس از آخرین جلسه تمرین نمونه خون از ورید باب جهت تعیین فعالیت آنزیم‌های پاراکسوناز-1، میلوپراکسیداز و سطح نیتریک اکسید در نمونه سرم تهیه شد. یافته­ ها: فعالیت آنزیم پاراکسوناز-1 سرم گروه‌های PPHE200 (47/1±02/23)، PPHE250 (98/1±59/23) و vitE (65/2±38/25) در مقایسه با گروه کنترل (38/1±57/18) افزایش معنی داری داشت (05/0P<). فعالیت آنزیم میلوپراکسیداز سرم گروه‌های PPHE200 (97/9±76/32)، PPHE250 (05/6±45/31) و vitE (56/4±94/24) در مقایسه با گروه کنترل (14/6±70/40) کاهش معنی داری داشت (05/0P<). سطح نیتریک اکسید سرم گروه‌های PPHE200 (48/2±59/46)، PPHE250 (62/2±27/40) و vit E (82/3±25/36) در مقایسه با گروه کنترل (36/5±18/47) کاهش معنی داری داشت (05/0P<). بحث و نتیجه ­گیری: نتایج تحقیق نشان داد مصرف عصاره پوست انار موجب بهبود وضعیت سیستم آنتی اکسیدانی، ضد التهابی و کاهش اثرات تنش اکسیداتیو ناشی از فعالیت شدید بدنی می‌گردد.

Title: مقایسه کارکردهای اجرایی در دانش‌آموزان با و بدون ناتوانیهای ویژه یادگیری همراه با مشخصه خواندن و نوشتن

http://yafte.lums.ac.ir/article-1-2408-fa.html

مقدمه: هدف این پژوهش بررسی کارکردهای اجرایی شامل حافظه کاری، سازمان­دهی-برنامه ­ریزی و استدلال در کودکان با و بدون ناتوانی‌های ویژه یادگیری همراه با مشخصه خواندن و نوشتن بود. مواد و روش ­ها: طرح این پژوهش پس­رویدادی بود و جامعه آماری کلیه دانش ­آموزان پسر پایه سوم مقطع ابتدایی با و بدون ناتوانی‌های ویژه یادگیری، در شهر تهران بود. نمونه پژوهش 90 دانش ­آموز پسر مقطع ابتدایی بود که در سه گروه 30 نفره با ناتوانی‌های ویژه یادگیری همراه با مشخصه خواندن، گروه با ناتوانی‌های ویژه یادگیری همراه با مشخصه اختلال نوشتن و گروه عادی بر اساس نمونه‌ گیری تصادفی سیستماتیک انتخاب شدند. ابزار گردآوری داده­ ها، خرده­ آزمون ­های حافظه رقمی و تشابهات وکسلر، آزمون­ آندره ری و آزمون غیررسمی خواندن و نوشتن بودند. داده ­ها با آزمون ANOVA تحلیل شدند. یافته ­ها: نتایج نشان داد که کودکان دارای ناتوانی‌های ویژه یادگیری در کارکردهای اجرایی حافظه کاری، سازمان­دهی-برنامه ­ریزی و استدلال با گروه عادی تفاوت داشتند (05/0 < P). همچنین بین دو گروه دارای ناتوانی‌های ویژه یادگیری همراه با مشخصه خواندن و نوشتن در حافظه کاری و استدلال تفاوت وجود داشت، ولی در مؤلفه سازمان­دهی-برنامه ­ریزی بین دو گروه دارای ناتوانی‌های ویژه یادگیری تفاوتی مشاهده نشد. بحث و نتیجه ­گیری: با توجه به این یافته ­ها، پیشنهاد می­شود برای دانش ­آموزان دارای ناتوانی‌های ویژه یادگیری، روش­هایی جهت بهبود حافظه کاری، سازمان­دهی-برنامه­ ریزی و استدلال اتخاذ شود.

Title: بررسی تأثیر ژنوتیپ‌های پلی‌مورفیسم -137G/C از ژن اینترلوکین 18 بر روی سطح ایمونوگلوبین E سرمی در بیماران مبتلا به رینیت آلرژیک

http://yafte.lums.ac.ir/article-1-2409-fa.html

مقدمه: بیماری رینیت آلرژیک به‌عنوان یک بیماری التهابی مزمن سیستم تنفسی است که می‌تواند تأثیر شدیدی بر روی کیفیت زندگی بیماران بگذارد. این پاسخ التهابی در ناحیه موکوسی لوله‌های هوایی شامل یک پاسخ با میانجی گری ایمونوگلوبین E به‌وسیله ماست سل‌ها است به‌طوری که واکنش‌های فاز تأخیری به‌واسطه ائوزینوفیل‌ها و بازوفیل‌ها انجام می‌شود. اینترلوکین 18 یک عضو از خانواده اینترلوکین‌ها است که به‌عنوان فاکتور القا کنندهIFN-γ  نامیده می‌شود و در تعادل واکنش‌های ایمنی مربوط به Th1/Th2  نقش دارد بنابراین هدف ما در این تحقیق بررسی ارتباط بین پلی‌مورفیسم-137G/C در پروموتر ژن اینترلوکین 18 با میزان IgE سرمی در مبتلایان بیماری رینیت آلرژیک بود. مواد و روش ­ها: سطح کلی IgE در سرم 131 بیمار مبتلا به رینیت آلرژیک و 62 فرد سالم به‌ روش الایزا اندازه گیری شد و سپس ارتباط سطح کلی IgE در افراد بیمار و سالم با پلی‌مورفیسم-137G/C مورد بررسی قرار گرفت. آنالیزهای آماری با نرم افزار 19SPSS  انجام گرفت. یافته­ ها: نتایج این تحقیق بیانگر عدم وجود ارتباط معنی دار بین ژنوتیپ‌های پلی­مورفیسم -137G/C و سطح IgE سرمی بین افراد بیمار مبتلا به رینیت آلرژیک و افراد سالم بود (05/0P>). بحث و نتیجه ­گیری: عدم وجود ارتباط معنی دار بین ژنوتیپ‌های پلی‌مورفیسم -137G/C ژن IL-18 با سطح IgE موجود در سرم بیانگر عدم نقش این ژنوتیپ در بروز بیماری رینیت آلرژیک می ­باشد.

Title: اثر محافظتی کوارستین بر روی ناهنجاریهای اسکلتی ناشی از دیابت تجربی در جنین موش صحرایی

http://yafte.lums.ac.ir/article-1-2410-fa.html

مقدمه: دیابت مادری می‌تواند ناهنجاری‌های تکاملی از جمله ناهنجاری‌های صورت و کام را ایجاد کند و این اثرات ناهنجاری زایی با مصرف داروهای آنتی اکسیدان کاهش می‌یابد. در این مطالعه اثر پیشگیری کننده کوارستین در وقوع ناهنجاری‌های جنینی ناشی از دیابت ارزیابی شد. مواد و روش ­ها: در این مطالعه تجربی از 24 سر موش صحرایی ماده ویستار در 4 گروه استفاده شد. پس از تزریق داخل وریدی 50 میلی گرم برای هر کیلوگرم وزن بدن استرپتوزوتوسین به دو گروه و تأیید دیابتی شدن، موش‌های همه گروه‌ها جفت گیری داده شدند. یک گروه دیابتی و یک گروه غیر دیابتی از موش‌ها، کوارستین را در روزهای صفر، 7، 14 و 20 آبستنی 75 میلی گرم بر کیلوگرم وزن بدن و گروه‌های دیگر سرم فیزیولوژی را به‌صورت خوراکی دریافت کردند. جنین‌ها در روز بیستم آبستنی جمع آوری شدند و بعد از تعیین طول و وزن و رنگ آمیزی، ناهنجاری‌های اسکلتی با استریو میکروسکوپ بررسی شد. یافته ­ها: درصد شکاف کام، دنده مواج و چسبیدن دنده در گروه کنترل دیابتی 09/38، 76/4 و 28/14 درصد بود ولی به‌وسیله کوارستین به ترتیب 12/16، صفر و صفر درصد کاهش یافت. همچنین کوارستین ناهنجاری‌های جناغ و دنده‌ها را کاهش داد. بحث و نتیجه­ گیری: کوارستین به‌عنوان یک ماده آنتی اکسیدان می‌تواند ناهنجاری‌های ناشی از دیابت شیرین را کاهش دهد.

Title: بررسی ارتباط بین کیفیت خواب و پیشرفت تحصیلی با سردردهای میگرنی در دانشجویان علوم پزشکی البرز

http://yafte.lums.ac.ir/article-1-2411-fa.html

مقدمه: توجه به مقوله کیفیت خواب در دانشجویان پزشکی به‌عنوان قشری از جامعه که با سلامت انسان‌ها سروکار دارند و رابطه آن با متغیرهای روانشناختی (خصوصاً پیشرفت تحصیلی و سردرد) ضروری به نظر می‌رسد. لذا پژوهش حاضر با هدف بررسی ارتباط بین کیفیت خواب و پیشرفت تحصیلی با سردردهای میگرنی مورد بررسی قرار گرفت. مواد و روش ­ها: پژوهش حاضر توصیفی و از نوع همبستگی بود. جامعه آماری پژوهش، شامل کلیه دانشجویان دانشگاه علوم پزشکی البرز در سال تحصیلی 93-92 بودند. با استفاده از روش نمونه گیری طبقه‌ای و طبق معیارهای ورود به پژوهش 256 نفر (156 نفر دختر و 100 نفر پسر) به‌عنوان نمونه انتخاب و به پرسشنامه کیفیت خواب و پرسشنامه سنجش علائم سردرد میگرن پاسخ دادند. همچنین معدل کل دانشجویان به‌عنوان شاخص پیشرفت تحصیلی در نظر گرفته شد. داده‌ها با استفاده از نرم افزار 19 SPSS تحلیل گردید. یافته ­ها: نتایج نشان داد سردردهای میگرنی با پیشرفت تحصیلی، کیفیت خواب کلی و زیر مقیاس‌های کیفیت ذهنی خواب، تأخیر در به خواب رفتن، اختلالات خواب، مصرف داروهای خواب آور و اختلال عملکرد روزانه همبستگی معنی داری دارند و اختلالات خواب، اختلال عملکرد روزانه، پیشرفت تحصیلی و مصرف داروهای خواب آور، 6/25% تغییرات مربوط به سردردهای میگرنی را پیش بینی کردند. بحث و نتیجه­ گیری: کیفیت خواب نامناسب در دانشجویان با بروز بیشتر سردردهای میگرنی و افت عملکرد تحصیلی در آنها همراه بود.

Title: امکان‌سنجی پیش‌بینی لیشمانیوز جلدی در پلدختر با استفاده از متغیرهای اقلیمی

http://yafte.lums.ac.ir/article-1-2412-fa.html

مقدمه: روند رو به افزایش مبتلایان به بیماری سالک در شهرستان پلدختر طی ده سال گذشته و پیشرفت تکنولوژی در تولید داده، ضرورت ساخت مدل‌های پیش‌بینی شیوع بیماری را در منطقه افزایش داده است. لذا در این بررسی از متغیرهای اقلیمی در پیش­بینی بیماری سالک استفاده شده است. مواد و روش ­ها: در این تحقیق با کمک مدل­ رگرسیونی رابطه بین آمار تعداد مبتلایان به بیماری و سیگنال‌های اقلیمی به‌صورت همزمان و با یک، دو، سه و چهار ماه تأخیر رگرسیون گرفته شده است. یافته ­ها: در مجموع بین بیماران بهمن با سیگنال اقلیمی nino1 دی، بیماران اسفند با سیگنال اقلیمی pna بهمن، بیماران فروردین با سیگنال اقلیمی AAMM اسفند، بیماران اردیبهشت با سیگنال اقلیمی AO فروردین و بیماران مرداد با سیگنال اقلیمی tsa خرداد، در سطح 5 درصد رابطه معنادار وجود داشت. همچنین بین بیماران بهمن با سیگنال اقلیمی nino1 دی در سطح 10 درصد رابطه معنادار وجود داشت. بحث و نتیجه ­گیری: این بررسی نشان داد که استفاده از سیگنال‌های اقلیمی با تأخیر برای پیش­بینی بیماری، بهتر از به‌کارگیری سیگنال­ها و بیماری به‌صورت همزمان است. بین آمار ابتلا به بیماری در کل دوره با سیگنال pdo با دو ماه تأخیر بیشترین میزان همبستگی (53/84 %) به دست آمد. علاوه بر این نتایج نشان داد که تقریباً در نیمی از ماه­ های سال همبستگی خوبی بین شیوع بیماری سالک و سیگنال‌های اقلیمی وجود دارد و درنتیجه می­توان با استفاده از سیگنال‌های اقلیمی به شیوع بیماری سالک پی برد.

Title: اثربخشی رویکرد هیجان ـ مدار بر تنظیم شناختی هیجان دانشجویان دختر دچار شکست عاطفی

http://yafte.lums.ac.ir/article-1-2416-fa.html

مقدمه: شکست عاطفی، حالت سرخوردگی و حقارت است که پس از طرد شدن توسط محبوب به فرد دست می ­دهد. لذا مطالعه حاضر باهدف بررسی اثربخشی رویکرد هیجان-­مدار بر تنظیم شناختی هیجان دانشجویان دختر دچار شکست عاطفی انجام شد. مواد و روش ­ها: طرح پژوهش نیمه آزمایشی از نوع پیش ­آزمون، پس ­آزمون و پیگیری با گروه گواه بود. جامه  ­ی آماری شامل کلیه دانشجویان دختر مبتلا به شکست عاطفی دانشگاه­ های شهر اهواز در سال 94-93 بود. حجم نمونه 22 نفر بود که از این تعداد، 11 نفر از آزمودنی ­هایی که رضایت کامل داشتند در گروه آزمایش و 11 نفر دیگر در گروه گواه جایگزین شدند. ابزارهای مورد استفاده شامل پرسشنامه ضربه عشق، پرسشنامه تنظیم شناختی هیجان بود. همه آزمودنی ­ها به دو پرسشنامه پاسخ دادند سپس افراد گروه آزمایش، مداخله درمانی را طی 8 جلسه 60 دقیقه ­ای به‌صورت فردی و 2 بار در هفته دریافت نمودند. در پایان هر دو گروه مجدداً به پرسش­نامه تنظیم شناختی هیجان پاسخ دادند. پس از گذشت یک ماه از اجرای پس ­آزمون، آزمون پیگیری برای هر دو گروه اجرا گردید. برای تجزیه و تحلیل داده ­ها علاوه بر آمار توصیفی، از روش آماری تحلیل کوواریانس چندمتغیری استفاده شد. یافته­ ها: یافته ­ها نشان داد رویکرد هیجان-­مدار در مرحله پس ­آزمون و پیگیری موجب استفاده بیشتر از راهبردهای مثبت شناختی تنظیم هیجان گروه آزمایش در مقایسه با گروه گواه شده ­است. بحث و نتیجه ­گیری: از آنجا که عشق و احساس ­های همراه با شکست عشقی از نوع هیجان است، لذا درمان هیجان-مدار می­تواند تأثیر معناداری بر تنظیم شناختی هیجان دانشجویان دختر دچار شکست عاطفی بگذارد.

Title: مطالعه اثر ضد سرطانی پروبیوتیک‌ها بر میزان پروتئین جهش یافته p53 در رده سلولی K562

http://yafte.lums.ac.ir/article-1-2417-fa.html

مقدمه: پروبیوتیک ­ها به‌عنوان میکروارگانیسم ­های گوناگونی تعریف می­شوند که دارای اثرات مفید در جلوگیری و درمان شرایط پاتولوژیک ویژه هستند. لاکتوباسیلوس کازیی و پاراکازیی، پروبیوتیک­هایی هستند که می­توانند آپوپتوز را در سلول­های سرطانی القاء کنند. لوسمی میلوییدی مزمن از شایع­ترین بدخیمی­ های بدن محسوب می­ شود. بیان پروتئین­ های جهش ­یافته p53 در رده­های سرطانی، مقاومت در مقابل آپوپتوز را افزایش می­دهند. بنابراین هدف از بررسی حاضر، ارزیابی تأثیر عصاره سیتوپلاسمی و دیواره سلولی این پروبیوتیک­ ها بر میزان پروتئین جهش‌یافته p53 در رده سرطانی K562 در شرایط آزمایشگاهی بود. مواد و روش ­ها: برای این منظور ابتدا باکتری­ها کشت داده شد، سپس به کمک دستگاه سونیکاتور خرد و درنهایت دیواره سلولی و عصاره سیتوپلاسمی آنها جدا شدند. در مرحله بعد غلظت­ های مختلفی از دیواره سلولی و عصاره سیتوپلاسمی تهیه شدند. سپس میزان پروتئین p53 در سلول‌های تیمار شده با عصاره سیتوپلاسمی و دیواره سلولی، به روش الایزا سنجیده شد. یافته­ ها: یافته ­های حاصل از آزمون الایزا نشان دادند، عصاره سیتوپلاسمی و دیواره سلولی باعث کاهش معنی­دار (05/0>P) سطح پروتئین حاصل از ژن جهش‌یافته p53 نسبت به گروه شاهد می­ شوند. بحث و نتیجه­ گیری: بنابراین می­توان نتیجه گرفت، بین کاهش میزان پروتئین p53 و القاء آپوپتوز در سلول­ها با ژن p53 جهش‌یافته رابطه وجود دارد که این می­تواند راهی برای درمان هدفمند سرطان ارائه کند.

Title: تأثیر عصاره هیدرو اتانولی صمغ درخت زردآلو بر روند بهبود زخم پوستی در موش صحرایی

http://yafte.lums.ac.ir/article-1-2418-fa.html

مقدمه: صمغ، در بهبود زخم در برخی بیماری‌ها و اختلالات مزمن یک چالش درمانی محسوب می‌شود. به لحاظ اثرات ضدالتهاب و ترمیمی عصاره هیدرو اتانولی صمغ درخت زردآلو، این عصاره در ترمیم زخم پوستی در موش صحرایی بررسی شد. مواد و روش ­ها: ۲۸ موش بزرگ نر نژاد ویستار به ۴ گروه تقسیم شدند. گروه اول: نرمال، گروه دوم: کنترل داری زخم بدون درمان، گروه سوم: دارای زخم درمان با وازلین و گروه چهارم: داری زخم درمان با عصاره صمغ درخت زردآلو. زخمی به ابعاد ۲۸۰ میلی‌متر بر پشت موش‌ها در گروه‌های ۲، ۳ و ۴ ایجاد شد. در گروه ۳ و ۴ به مدت ۲۱ روز درمان ادامه یافت. تجویز داروها به‌صورت روزانه و به مقدار نیم گرم بر روی زخم به کار رفت. اندازه‌گیری یک روز در میان اندازه زخم‌ها با آنالیز تصاویر دیجیتال به مدت ۲۱ روز انجام گرفت. نمونه‌گیری از پوست تمام حیوانات در روز ۲۱ انجام و نمونه در فرمالین فیکس شدند. برش‌ها به ضخامت ۵ میکرون تهیه و با هماتوکسیلین- ائوزین رنگ‌آمیزی شدند. برش‌های بافتی مورد بررسی کیفی و کمی برای بررسی قطر پوشش، میزان رشته‌های کلاژن، عروق و سلول‌های آماسی مطالعه شدند. داده‌های نرمال، با استفاده از آزمون توکی و داده‌های با توزیع غیر نرمال با آزمون من ویتنی بین گروه‌ها مقایسه شدند. یافته ­ها: استفاده از صمغ درخت زردآلو، باعث کاهش عروق خونی از هفته دوم به بعد، کاهش سلول‌های آماسی و افزایش رشته‌های کلاژن و درنهایت اپیتلیزاسیون بافت شد. بحث و نتیجه ­گیری: استفاده از عصاره صمغ درخت زردآلو می‌تواند ترمیم زخم را سریع‌تر بهبود بخشد.

Title: تأثیر یک دوره تمرین هوازی و یک دوره بی تمرینی پس از آن بر فشارخون استراحت دانشجویان دختر غیر ورزشکار شهرستان خرم‌آباد

http://yafte.lums.ac.ir/article-1-2419-fa.html

مقدمه: فعالیت ورزشی نقش مهمی در ارتقای عملکرد قلب و عروق دارد. هدف تحقیق حاضر تعیین تأثیر 10 هفته تمرین هوازی و 6 هفته بی تمرینی بر فشارخون استراحت دانشجویان دختر سالم غیر ورزشکار بود. مواد و روش ­ها: در این تحقیق نیمه تجربی 27 دانشجوی دختر غیر ورزشکار با میانگین سنی 7/1 ± 30/22 به‌صورت داوطلبانه انتخاب شدند و در برنامه تمرینی 10 هفته‌ای، هفته‌ای 3 جلسه با شدت 55 تا 80 درصد ضربان قلب بیشینه شرکت کردند. پس از 10 هفته تمرین هوازی، افراد مورد مطالعه 6 هفته در بی تمرینی به سر بردند. قبل از شروع دوره تمرین هوازی، پس از دوره تمرین و همچنین پس از دوره بی تمرینی فشارخون استراحت افراد مورد مطالعه، به‌وسیله فشارسنج جیوه‌ای مدل ریشتر اندازه‌گیری شد. یافته ­ها: نتایج نشان داد که بین فشارخون سیستول استراحت افراد مورد مطالعه در پیش آزمون و پس آزمون 1 و همچنین در پس آزمون 1 و پس آزمون 2 اختلاف معنی‌داری وجود دارد، ولی در فشارخون دیاستول استراحت افراد مورد مطالعه در این آزمون‌ها اختلاف معنی‌داری وجود ندارد. بحث و نتیجه ­گیری: 10 هفته تمرین هوازی و 6 هفته بی تمرینی پس از آن می‌تواند بر فشارخون استراحت مؤثر باشد.
